# Optimising, generalising and integrating educational practice using neuroscience

**DOI:** 10.1038/npjscilearn.2016.12

**Published:** 2016-07-06

**Authors:** Robert Colvin

**Affiliations:** 1 School of Information Technology and Electrical Engineering, University of Queensland, Brisbane, QLD, Australia

## Abstract

Practical collaboration at the intersection of education and neuroscience research is difficult because the combined discipline encompasses both the activity of microscopic neurons and the complex social interactions of teachers and students in a classroom. Taking a pragmatic view, this paper discusses three education objectives to which neuroscience can be effectively applied: optimising, generalising and integrating instructional techniques. These objectives are characterised by: (1) being of practical importance; (2) building on existing education and cognitive research; and (3) being infeasible to address based on behavioural experiments alone. The focus of the neuroscientific aspect of collaborative research should be on the activity of the brain before, during and after learning a task, as opposed to performance of a task. The objectives are informed by literature that highlights possible pitfalls with educational neuroscience research, and are described with respect to the static and dynamic aspects of brain physiology that can be measured by current technology.

## Introduction

Our understanding of the brain has sufficiently advanced to enable cross-disciplinary collaboration on learning.^1–3^ However, there are still significant limits to neuroscientific knowledge, due to the complexity of the brain itself and the technological and financial constraints of current tools. Furthermore, there are many potential pitfalls in conducting educational neuroscience research.^4,5^ Taking these issues into account, this paper argues that the most effective avenue for cross-disciplinary research into improving student attainment lies in applying neuroscience to the three aims of optimisation, generalisation and integration of educational interventions (instructional techniques, interpreted broadly).

**Figure 1:**
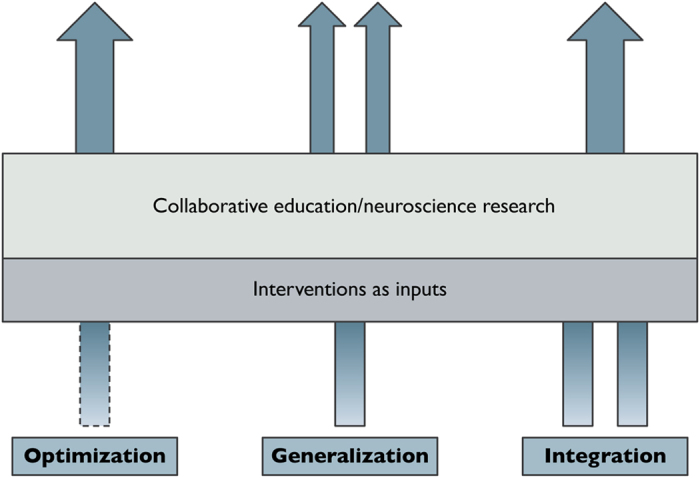
Existing instructional techniques are here viewed as ‘inputs’ to educational neuroscience research. A particular technique may be optimised by determining the combination of parameters (length of study session and timing of exams) that is in harmony with neural processes. Alternatively, a particular intervention may be generalised to different contexts or domains, based on the attributes of the neural processes underlying the learning of a particular skill. Finally, two or more interventions, perhaps drawn from diverse research, may be combined into a single intervention, resulting in a technique that is optimal from both a within-lesson attention perspective as well as long-term retention of memories.

The three aims are depicted in Figure 1.

- Optimisation refers to improving an existing intervention to achieve maximal results.
- Generalisation refers to adapting an effective intervention in one domain for application in a different domain or setting (also called transfer).
- Integration refers to taking one or more complementary techniques and combining them into a more comprehensive intervention.

These aims are related in that an understanding of the underlying biology can have a direct role in identifying improvements in existing practice, and furthermore, in general no finite number of behavioural experiments alone can address them. Of course, any hypotheses based on biological processes must be validated by behavioural experiments. Crucially, we argue that research should be conducted by looking at the changes in brain activity over days or weeks as a result of learning interventions. This is in contrast to many treatments, which assumes that neuroscientific input is limited simply to determining the regions of the brain an expert uses to complete a task. We outline below the structure and dynamics of the brain from the perspective of relating performance to educational attainment.

## The brain: a summary and its relationship with academic abilities

At the anatomical level, the brain is often regarded as a network of regions (grey matter) connected by neural tracts (white matter). The individual regions of the brain show specialisation for particular tasks, such as visual processing, numeric processing, etc. Many real-world activities involve a combination of such tasks and hence require the integration of neural activity (e.g., externally driven aural input being combined with internally driven memories), and this information from different parts of the brain is transmitted by the white matter connections. For a complex task that takes more than a few hundred milliseconds and involves multiple types of sensory input, the activity of the brain may be temporally staged, where the activity takes place mostly in sensory processing areas before becoming more dominant in areas associated with higher-order cognitive abilities. We call such patterns of activity functional activation signatures. The performance of cognitive tasks emerges from such activity.

Any learning—that is, a change in behaviour or new knowledge between time 1 and time 2—indicates a physical change in the brain. This may be a change in synaptic strength (called synaptic plasticity, e.g., Feldman^6^), or through the growth of new neurons (neurogenesis, e.g., Murphy *et al.*^7^ and Vukovic *et al.*^8^), or changes in the neurons themselves (e.g., Buonomano and Merzenich^9^). Changes may take place in the grey matter, the white matter or both. Any change is stimulated by the activity of neurons engaged in the learning task, which could be in the order of billions. The cumulative changes at the neural level may be evident at the anatomical level through improvements in processing speed within regions or communication speed between regions.

**Figure 2:**
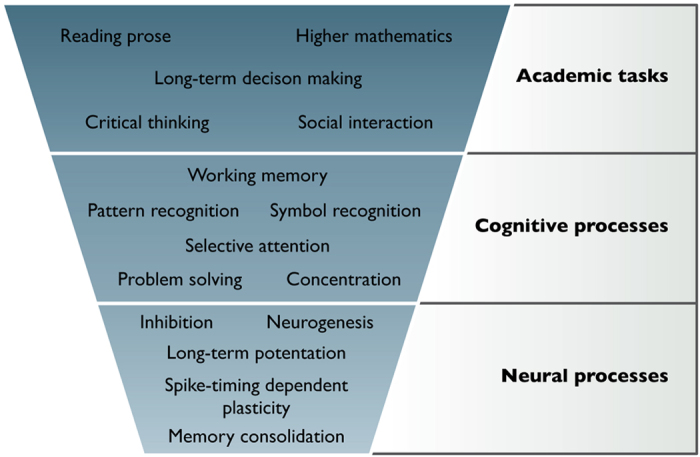
A depiction of how neural processes are composed to generate high-level academic abilities. At the bottom-most level are neurons and processes in which they are directly involved. Emerging from the interaction of these basic elements are higher-level (but still abstract) cognitive processes. These processes are applied in combination to tackle academic problems.

Figure 2 contains an incomplete classification of mental processes. ‘Neural processes’ refer to the basic infrastructure and subconscious processes of the brain, and are amenable to direct analysis using neuroscientific methods. Typically, multiple neural processes may be involved in ‘cognitive processes’, which are in a sense the basic units of conscious cognition, such as selective attention, symbol processing, working memory, pattern recognition and abstract problem solving. These skills may in turn be composed into ‘academic abilities’, such as critical reading, writing, higher mathematics, social interactions, long-term planning and decision-making, etc. The distinction between skills and processes through the three levels is of course arguable in many cases, but is designed to highlight that academic abilities are ultimately formed from underlying neural processes.

## Building on biological data

Cognitive performance is usually behaviourally measured by both accuracy and reaction time. The progression from novice to expert is indicated by an improvement in one or both measures. A significant change in reaction time correlates by definition with a change in the activation signature, perhaps highlighting where neural bottlenecks have been bypassed^10^ or consolidation from conscious to subconscious processing regions has occurred.^11,12^ The application of such data to the three objectives is outlined below, illustrated using a hypothetical intervention for teaching a geometry task.

1. *Optimisation.* A comparison of activation signatures between novice and expert on a task may show that the same regions are activated in the same order, but with shorter (and/or longer) times spent in each. Alternatively, experts may show activation in other regions, perhaps as the result of automation or better cognitive techniques. This information may be used to adapt an intervention to focus on the acquisition of the relevant factors of expert performance as distinct from novice performance. In the hypothetical geometry task, the changes in activation signatures may suggest novices experience bottlenecks in the visual processing region of the brain. The intervention may be refocused based on those observations to change the level of visual stimulus or to split the task into visual and non-visual subcomponents. In such cases, bypassing processing bottlenecks may result in quicker mastery of the topic.
2. *Generalisation.* Comparing functional activation signatures across tasks can lead to generalisation by finding related patterns in terms of timing and regions involved. That is, if an intervention improves proficiency in a task whose signature resembles or overlaps with that of another task, it is reasonable to investigate whether the benefits of the intervention transfer to the other domain. For instance, it may be the case that proficiency in a grammatical task is attained when a similar visual processing bottleneck is bypassed as in the geometry task. Adapting the grammatical intervention to split tasks into visual and non-visual components along the same lines as the geometry intervention may provide the same benefit to learning.
3. *Integration.* Comparison of how signatures develop may identify harmonious interventions, and concretely suggest ways in which they can be combined. The integration may be task-specific, or more general in the sense of combining task-specific considerations with approaches to structuring lessons to align with long-term memory storage. For instance, if the signature for a second successful intervention for the same geometry task is found to emphasise processing speed in the visual areas, an integrated intervention may compound the benefits of both. Furthermore, when the intervention is considered as a programme taking place over several weeks, the improvements in the spatial element of the task may occur from leaving longer gaps between interventions^13^ and incorporating extra testing.^14^

There is no guarantee that any of the potential outcomes from collecting activation signatures will eventuate, as both the external environment and the internal workings of the brain are complex; however, the path to addressing the three objectives is more rigorous in comparison with hypotheses based on behaviour alone. Furthermore, harmonies between interventions may be found that would not otherwise reveal themselves without an understanding of the underlying signatures.

## Criticisms of educational neuroscience

A significant impediment to the use of neuroscience in education is that much neuroscientific knowledge is far removed from classroom interactions (a ‘bridge too far’^4^). As such care must be taken in the choice of intervention and the framing of the corresponding neuroscientific investigation, and cognitive psychology research must be incorporated. Fortunately, As neuroscience research and technology continues to advance the gap will lessen. An argument that neuroscience has no value to education research appears weak, as that would imply that studies involving the integration of educational, psychology and neuroscience research would be made stronger by removing the neuroscience component. It is to be hoped that in the future the distinction between the three areas with respect to learning^15^ will be seen as archaic.

There are at least two important criticisms of educational neuroscience that have informed the current proposal: (1) some facets of neuroscience are not relevant to some facets of classroom learning; and (2) research into learning disorders such as dyslexia^16,17^ and dyscalculia^18^ have relatively less relevance to typical classroom situations. This paper expands on arguments for educational neuroscience,^19–21^ ameliorating the above problems by providing a pragmatic framework for applying neuroscience to existing educational research.

## Conclusion

Effective smallpox vaccines were developed through observation; over the subsequent centuries, an understanding of why the vaccines were effective lead to optimisations of delivery and generalisation to the treatment of other diseases. Experience will continue to have the leading role in education research, but the input from neuroscience provides an opportunity to increase the scope and benefit of effective interventions. This flows naturally from understanding why something is effective, in addition to what is effective.

Neuroscience research will have greatest impact when it builds upon the wealth of existing education research into effective learning,^22^ provided the right questions are addressed. Here we argue for a narrow focus on optimising, generalising and integrating existing interventions at the cognitive level. Neuroscience is unlikely to directly impact on other important aspects of student attainment and education, such as teacher career paths, governmental policy or curriculum. The organisation and function of the brain may have relevance to questions of class size, for instance, but such considerations will always need to be balanced against financial and administrative realities. And, any intervention must be sensitive to the social and cultural aspects of a particular learning environment.

Where neuroscience can provide otherwise difficult-to-obtain data is in the details of the progression from novice to expert. Expertise may be obtained in a variety ways; brain activity provides a measure of temporal and spatial changes that underlie successful (or unsuccessful) educational attainment that is more fine-grained than that achievable by behavioural testing alone.

There are many promising avenues to which neuroscience may be applied. Research such as Hattie’s analysis^23^ of techniques that influence learning are ideal starting places for deeper research into effective teaching interventions. One interpretation of that research is that the most powerful results are achieved when students see themselves as active participants in learning and have clearly defined goals. This result appears to generalise across domains, and would seem to integrate with other, more domain-specific approaches to teaching. To build on such insights with the intention of achieving maximum results, wide applicability and the best of all worlds, the complex social activity of humans learning complex topics in real environments^24–26^ must be linked to the fundamental processes that result in physical changes in the brain as it learns.
